# The MORPHEUS protein crystallization screen

**DOI:** 10.1107/S0021889809042022

**Published:** 2009-11-07

**Authors:** Fabrice Gorrec

**Affiliations:** a MRC Laboratory of Molecular Biology, Hills Road, Cambridge CB2 0QH, England

**Keywords:** macromolecular crystallography, macromolecular crystallization, crystallization screening, crystallization additives

## Abstract

A 96-condition initial screen for protein crystallization, called MORPHEUS, has been developed at the MRC Laboratory of Molecular Biology, Cambridge, England (MRC-LMB). The concept integrates several innovative approaches, such as chemically compatible mixes of potential ligands, new buffer systems and precipitant mixes that also act as cryoprotectants. Instead of gathering a set of crystallization conditions that have already been successful, a selection of molecules frequently observed in the Protein Data Bank (PDB) to co-crystallize with proteins has been made. These have been put together in mixes of similar chemical behaviour and structure, and combined with buffers and precipitant mixes that were also derived from PDB searches, to build the screen *de novo*. Observations made at the MRC-LMB and many practical aspects were also taken into account when formulating the screen. The resulting screen is easy to use, comprehensive yet small, and has already yielded a list of crystallization hits using both known and novel samples. As an indicator of success, the screen has now become one of the standard screens used routinely at the MRC-LMB when searching initial crystallization conditions for biological macromolecules.

## Abstract 2

MORPHEUS is an initial protein crystallization screen with a unique organization which integrates components and ligands selected after analysing all crystal structure data deposited with the Protein Data Bank and local data gathered at the MRC Laboratory of Molecular Biology, Cambridge, England (MRC-LMB). Three challenging proteins from the MRC-LMB have already been crystallized exclusively using MORPHEUS.

## 1. Introduction   

Structure determination of biological macromolecules has been tremendously successful over recent years. The Protein Data Bank (PDB, http://www.pdb.org; Berman *et al.*, 2000 ▶) now holds nearly 60 000 coordinate sets. Approximately 80% of those have been determined by X-ray crystallography, and the method, since its first application to biological macromolecules more than 50 years ago (Kendrew *et al.*, 1958 ▶; Perutz *et al.*, 1960 ▶), has continued to improve. Recently, the atomic structure of the complete 70S ribosome was determined using X-ray crystallography (Selmer *et al.*, 2006 ▶). Given the obvious successes, one might be forgiven for assuming that the basis of the method, the crystallization of a protein, DNA or RNA and their complexes, must be an easy process. In fact, crystallization is now rate limiting and a typical project trying to elucidate the structure of a biological macromolecule of interest will spend most time trying to obtain a sample of biological interest that can be crystallized (Chayen & Saridakis, 2008 ▶). The underlying problem is that at the time of the crystallization experiment the structure of the molecule is not known and hence a rational approach cannot be taken.

To circumvent this problem, crystallization screens are utilized which try to sample the vast number of possible variables in a manageable and efficient way, either systematically or randomly (McPherson, 2004 ▶). Development of an effective search strategy depends on determining how parameter variations influence crystal formation and crystal quality (Kingston *et al.*, 1994 ▶). The protein itself can be considered as the main variable (Dale *et al.*, 2003 ▶). However, the correct composition of the initial crystallization screen is necessary, although by no means sufficient, for success.

Nowadays, vapour diffusion with 50–200 nl drops is the most widespread crystallization technique and many different commercial screening kits are available to initiate experiments (Berry *et al.*, 2006 ▶). Many screens are systematic variations of the concentrations or chemical nature of the components and others employ so-called sparse-matrix approaches that are essentially collections of conditions (mixes of reagents used for protein crystallization) that have been found to work previously with other samples (Jancarik & Kim, 1991 ▶).

The increasing number of structures deposited in the PDB has motivated some statistical analyses of the crystallization conditions employed (Hennessy *et al.*, 2000 ▶; Kantardjieff & Rupp, 2004 ▶), together with attempts to rationalize protein crystallization screens (Zhu *et al.*, 2006 ▶; Newstead *et al.*, 2008 ▶). Rationalization has led to screens with a minimal number of conditions in sparse matrices and footprint screens (Brzozowski & Walton, 2001 ▶; Radaev & Sun, 2002 ▶; Tran *et al.*, 2004 ▶; Newman *et al.*, 2005 ▶). This is logical if overall efficiency is the main goal, such as in structural genomics.

At the MRC Laboratory of Molecular Biology (Cambridge, England), protein samples, DNA–protein complexes and RNA-containing complexes are regularly screened using standard procedures with more than 40 commercial initial screen kits (Stock *et al.*, 2005 ▶) and over 1500 conditions, assembled into pre-filled MRC 96-well crystallization plates. This large number is still not large enough because many samples fail to crystallize or give only a very few hits. Amongst others, this could be due to two main reasons. Firstly, the vast number of possible conditions is under-sampled (which is surely true). Secondly, crystallization can be critically dependent on the component(s) in the screen (St John *et al.*, 2008 ▶) that make proteins behave differently (more stable or rigid, for example). The latter reason is the rationale behind classical additive screening (Cudney *et al.*, 1994 ▶) and a recent development called Silverbullets (McPherson & Cudney, 2006 ▶).

Both assumptions were a driving force behind my attempts to formulate the new screen MORPHEUS that could enhance the chances of crystallization. The most important feature of MORPHEUS is the inclusion of mixes containing potential ligands and additives that can promote crystallization through specific interactions. This strategy includes the risk that one component of a mix might have a deleterious effect on crystal growth (or complex association) and thereby mask the positive contribution of another (Larson *et al.*, 2007 ▶). By selecting components that have been seen to be ordered in crystal structures in the PDB, the chances of incorporating molecules playing a positive role should increase.

An extensive search of the PDB was performed and small molecules and ions that bind to biological macromolecules were selected. The molecules are stable, commercially available, have a molecular weight below 250 Da and are easy to handle. Components found abundantly in the PDB are potentially good crystallization agents for two reasons. Firstly, they can be stabilizers. For example, some sugars are well known for their thermodynamic stabilization of macromolecules (Arakawa & Timasheff, 1982 ▶). Stabilization can also mean ‘rigidifying’ the protein or the crystal lattice and thus improving diffraction quality. Secondly, ligands can create crystallization variants by changing possible interactions on the molecular surface, hence increasing the chances of obtaining different crystals. From this perspective, small counter-anions like nitrate, phosphate and sulfate, with a multitude of possible binding modes *via* different spatial arrangements of O atoms, are ideal components. For the same reason, small organic salts with carboxylic acid groups can facilitate crystal growth (McPherson, 2001 ▶). Additional agents found frequently in the PDB include halides that promote different crystal forms (Lim *et al.*, 1998 ▶) and can help with crystallographic phase determination (Dauter *et al.*, 2000 ▶). It has been shown that polyethylene glycols (PEGs) tend to form linear binding patterns in clefts on protein surfaces (Hasek, 2006 ▶). Therefore, a selection of six PEGs completes the formulation of MORPHEUS.

MORPHEUS provides 96 original conditions made from innovative mixes of potential ligands that have been found with high frequency in the PDB. Will MORPHEUS, like the Greek god of dreams, take different forms, especially those in the shape of crystals? Here, ideas about the formulations and the results from crystallization experiments using test proteins and novel samples are described, proving the high usability and efficiency of MORPHEUS.

## 2. Materials and methods   

**Figure 1:**
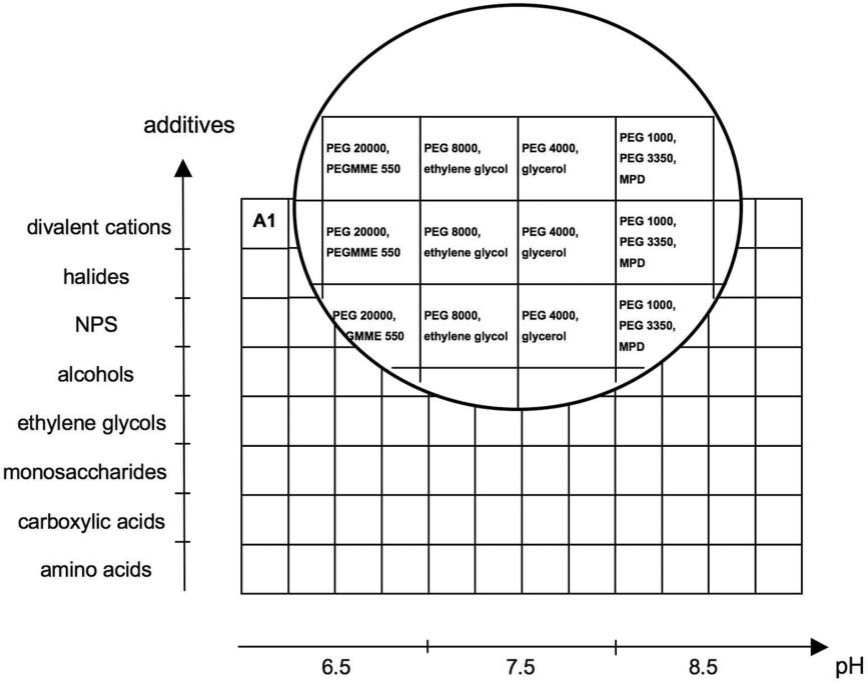
MORPHEUS schematic screen layout.

The complete formulation of MORPHEUS is shown in Table 1 ▶. Fig. 1 ▶ is a schematic representation of the screen layout.

### 2.1. Selection of PDB-derived ligands   

The set of 47 PDB-derived ligands is listed in Table 2 ▶. Initially, structures with ligand(s) were tabulated (July, 2008). Data were then filtered with a molecular weight cut-off of 250 Da. The resulting list was filtered again to keep only ligands seen with at least five unrelated protein structures.

Not included in MORPHEUS because of chemical incompatibility are all phenols, heavy atoms and detergents. Many divalent cations and some carboxylic acids were discarded in later tests because of problems with stability and false positives. Also, there is a limit to the number of ligands (*i.e.* additives) that can be integrated into 96 conditions. Concentrations must be high because low affinities should be considered (Sauter *et al.*, 1999 ▶).

### 2.2. Additive mixes   

Thirty-eight of the selected PDB-derived ligands have been grouped into families depending on their chemical nature to form eight additive mixes. For example, one of the additive mixes is composed of *n*-ethylene glycols (*n* = 2–5). By grouping the additives based on chemical nature, the possibility of cross-reaction is avoided and stock solutions are stable. When additives were salts with an acid or base form, the salts were selected so that the final pH of the mix was as neutral as possible. A compound-to-protein ratio of 10:1 is commonly adopted for co-crystallization with small molecule ligands (Danley, 2006 ▶) and hence the final concentration of each additive in MORPHEUS is 0.02 *M* minimum, representing ten times the concentration of a 10 kDa protein at 20 mg ml^−1^. The recipes for preparing the eight MORPHEUS additive mixes can be found in Table 3 ▶.

### 2.3. Precipitant mixes   

Precipitants can be mixed to have a synergistic effect (Majeed *et al.*, 2003 ▶) and/or to provide cryoprotection (Mitchell & Garman, 1994 ▶; McFerrin & Snell, 2002 ▶). To take advantage of these findings, four precipitant mixes were integrated in the formulation of MORPHEUS. Three of the mixes have been observed to be more successful in the crystallization of MRC-LMB samples than expected from their under-sampling in our initial screens, as described previously. A fourth mix was designed from scratch with components not found in the other three mixes. Principally, the precipitant mixes have been chosen so that the final conditions produce vitrified ice when frozen. It should be noted, however, that the optimal concentration of cryoprotectant is sample dependent and may need optimization later (Chinte *et al.*, 2005 ▶). Recipes for preparing the four MORPHEUS stock solutions with precipitants can be found in Table 4 ▶. The table includes the frequency of similar mixes in our MRC-LMB standard initial screens.

### 2.4. Buffer systems   

Six of the selected PDB-derived ligands described before have been used to build three buffer systems within a physiological pH range, namely 6.5, 7.5 and 8.5. The common advantage of buffer systems is that no titration with concentrated acid or base is required (Newman, 2004 ▶). Each MORPHEUS buffer system includes an acid and base pair of buffers with similar p*K_a_* values. This way, the systems combine the characteristics of two different Good buffers for biological research (Good *et al.*, 1966 ▶). 

Recipes for preparing 50 ml of the three MORPHEUS buffer systems can be found in Table 5 ▶. Non-titrated stock solutions of the individual buffers (at a concentration of 1 *M*) were mixed at different ratios for optimization purposes.

The chemicals used for making the buffer systems were MES [2-(*N*-morpholino)ethanesulfonic acid; Sigma, M8250, pH 2.7], imidazole (1,3-diazacyclopenta-2,4-diene; BDH, 286874D, pH 9.9), MOPS [3-(*N*-morpholino)propanesulfonic acid; BDH, 4438321, pH 2.9], HEPES-Na [sodium 4-(2-hydroxy­ethyl)piperazine-1-ethanesulfonate; Melford, B2001, pH 10.4], bicine [*N*,*N*-bis(2-hydroxyethyl)glycine; Fluka, 14871, pH 4.9] and Trizma base [proprietary Tris, 2-amino-2-(hydroxy­methyl)-1,3-propanediol; Sigma, T1503, pH 10.6]. The pH was measured at 294 K with an InLab 490 solid-state probe (Mettler–Toledo) to avoid inaccuracies with Tris-containing buffers.

### 2.5. Stability tests   

The stability of the conditions during their development was assessed by checking the turbidity and pH after one week at 293 K, one week at 277 K and another week at 293 K.

### 2.6. Proteins   

For details of the proteins used, please refer to Table 6 ▶.

### 2.7. Crystallization trials   

MRC crystallization plates (Swissci) containing MORPHEUS (85 µl in the main wells) were prepared on a Mosquito (TTP labtech) or ScreenMaker (Innovadyne) nanolitre liquid handler. Our standard setup for initial screens is to mix equal-volume aliquots of the protein and condition at 297 K, with a 200 nl final volume of drops, and to store the plates at 292 K. Final assessments were made after one week by manual inspection using a high-powered Leica MX-12 stereomicroscope. A drop was considered a crystallization hit when it contained protein crystals larger than 20 µm, so that they could be mounted in a cryoloop for X-ray diffraction.

### 2.8. Optimization of conditions   

Finally, all three components, the ligand mixes, the precipitant mixes and the buffers, are combined using a fixed ratio,
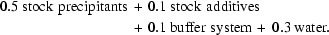
This simple recipe facilitates easy follow-up optimization experiments. As an initial approach, one can simply change the above ratios of the stock solutions. The composition of the buffer systems may be altered during optimization experiments to change the pH. Obviously, all of these optimization experiments are very amenable to automation (Hennessy *et al.*, 2009 ▶).

## 3. Results and discussion   

**Figure 2:**
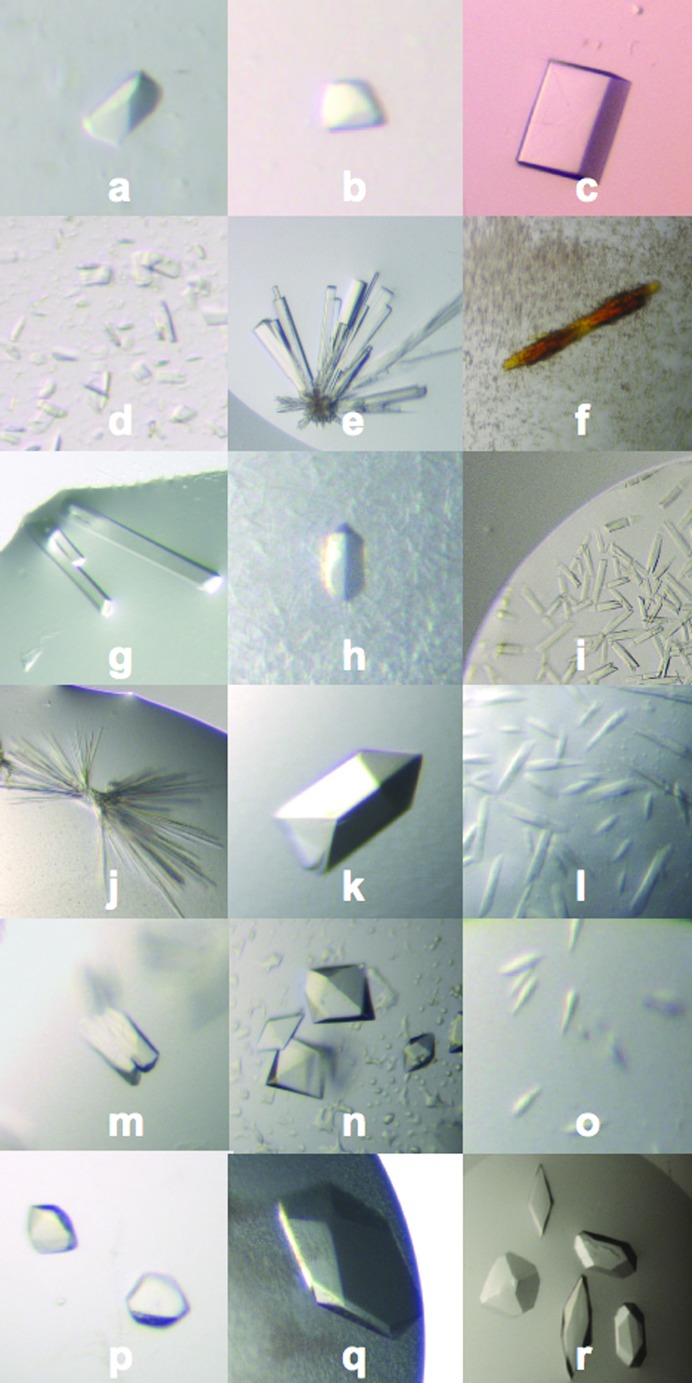
Light micrographs showing 18 crystals obtained with MORPHEUS (letters refer to Table 6 ▶, last column). Magnifications differ and crystal sizes vary between 20 and 600 µm.

Both well known test proteins and novel samples were tried with MORPHEUS. Table 6 ▶ shows all the details and results of the crystallization trials performed for 16 samples. Fig. 2 ▶ shows the different crystal morphologies observed. All the crystals shown represent initial hits, except for Scc3 (domain of sister chromatid cohesion protein 3) and PI3K-I (pi3-kinase p110 in complex with isoform-specific inhibitors) which involved optimization.

Importantly, three samples have crystallized exclusively in MORPHEUS and produced no hits from any other screen tried (over 1500 conditions): Scc3, PI3K-I and TriUb-D (triubiquitin in complex with a ubiquitin-binding domain).

The possible specificity of ligand mixes can be spotted easily because of the systematic screen layout: when there are several hits in the same row of MORPHEUS, it means there is specificity to ligands used in the conditions of that row (see samples PI3K-I, ParR, PAK4G and THM). In the same way, specificity to precipitant(s) and pH can easily be noticed (see Fig. 1 ▶). For example, most of the hits with the test sample BAR were in conditions that integrate the mix of precipitants developed for MORPHEUS (mix found in columns 4, 8 and 12: 12.5% PEG 1000, 12.5% PEG 3350 and 12.5% MPD).

## 4. Conclusions   

The advantages of designing an initial screen *de novo* have been demonstrated. MORPHEUS delivers a screen that is easy to make and the conditions are easy to optimize. It contains components that have been selected from crystallized complexes of previously published structures. It also contains a limited number of precipitant mixes that have been selected using local data from the MRC-LMB. MORPHEUS has been successful in crystallizing both known proteins and important new samples.

Ideally, more small molecules with interesting characteristics that are not used in commercially available screens should be investigated, like some polyols (Cohen *et al.*, 1993 ▶). An extensive set of amine derivatives, including well known polyamine additives (Ding *et al.*, 1999 ▶) and aminated amino acids (Matsuoka *et al.*, 2007 ▶), could form an excellent additive screen with frozen solutions for storage. Also, protein chaperones could be added for some challenging crystallizations (Ostermeier *et al.*, 1995 ▶; Tereshko *et al.*, 2008 ▶). In the same spirit, it would be interesting to investigate what could be done with molecules designed to mimic protein–protein interactions (Allen *et al.*, 1998 ▶).

## Figures and Tables

**Table 1:** Formulation of MORPHEUS PEG MME is polyethylene glycol monomethyl ether. MPD is (*RS*)-2-methyl-2,4-pentanediol. NPS is a mix containing sodium nitrate, disodium hydrogen phosphate and ammonium sulfate.

Well	Mix of precipitants	Mix of additives	Buffer system
A1	10% *w*/*v* PEG 20000, 20% *v*/*v* PEG MME 550	0.03*M* of each divalent cation	0.1*M* MES/imidazole pH6.5
A2	10% *w*/*v* PEG 8000, 20% *v*/*v* ethylene glycol	0.03*M* of each divalent cation	0.1*M* MES/imidazole pH6.5
A3	10% *w*/*v* PEG 4000, 20% *v*/*v* glycerol	0.03*M* of each divalent cation	0.1*M* MES/imidazole pH6.5
A4	12.5% *w*/*v* PEG 1000, 12.5% *w*/*v* PEG 3350, 12.5% *v*/*v* MPD	0.03*M* of each divalent cation	0.1*M* MES/imidazole pH6.5
A5	10% *w*/*v* PEG 20000, 20% *v*/*v* PEG MME 550	0.03*M* of each divalent cation	0.1*M* MOPS/HEPES-Na pH7.5
A6	10% *w*/*v* PEG 8000, 20% *v*/*v* ethylene glycol	0.03*M* of each divalent cation	0.1*M* MOPS/HEPES-Na pH7.5
A7	10% *w*/*v* PEG 4000, 20% *v*/*v* glycerol	0.03*M* of each divalent cation	0.1*M* MOPS/HEPES-Na pH7.5
A8	12.5% *w*/*v* PEG 1000, 12.5% *w*/*v* PEG 3350, 12.5% *v*/*v* MPD	0.03*M* of each divalent cation	0.1*M* MOPS/HEPES-Na pH7.5
A9	10% *w*/*v* PEG 20000, 20% *v*/*v* PEG MME 550	0.03*M* of each divalent cation	0.1*M* bicine/Trizma base pH8.5
A10	10% *w*/*v* PEG 8000, 20% *v*/*v* ethylene glycol	0.03*M* of each divalent cation	0.1*M* bicine/Trizma base pH8.5
A11	10% *w*/*v* PEG 4000, 20% *v*/*v* glycerol	0.03*M* of each divalent cation	0.1*M* bicine/Trizma base pH8.5
A12	12.5% *w*/*v* PEG 1000, 12.5% *w*/*v* PEG 3350, 12.5% *v*/*v* MPD	0.03*M* of each divalent cation	0.1*M* bicine/Trizma base pH8.5
B1	10% *w*/*v* PEG 20000, 20% *v*/*v* PEG MME 550	0.03*M* of each halide	0.1*M* MES/imidazole pH6.5
B2	10% *w*/*v* PEG 8000, 20% *v*/*v* ethylene glycol	0.03*M* of each halide	0.1*M* MES/imidazole pH6.5
B3	10% *w*/*v* PEG 4000, 20% *v*/*v* glycerol	0.03*M* of each halide	0.1*M* MES/imidazole pH6.5
B4	12.5% *w*/*v* PEG 1000, 12.5% *w*/*v* PEG 3350, 12.5% *v*/*v* MPD	0.03*M* of each halide	0.1*M* MES/imidazole pH6.5
B5	10% *w*/*v* PEG 20000, 20% *v*/*v* PEG MME 550	0.03*M* of each halide	0.1*M* MOPS/HEPES-Na pH7.5
B6	10% *w*/*v* PEG 8000, 20% *v*/*v* ethylene glycol	0.03*M* of each halide	0.1*M* MOPS/HEPES-Na pH7.5
B7	10% *w*/*v* PEG 4000, 20% *v*/*v* glycerol	0.03*M* of each halide	0.1*M* MOPS/HEPES-Na pH7.5
B8	12.5% *w*/*v* PEG 1000, 12.5% *w*/*v* PEG 3350, 12.5% *v*/*v* MPD	0.03*M* of each halide	0.1*M* MOPS/HEPES-Na pH7.5
B9	10% *w*/*v* PEG 20000, 20% *v*/*v* PEG MME 550	0.03*M* of each halide	0.1*M* bicine/Trizma base pH8.5
B10	10% *w*/*v* PEG 8000, 20% *v*/*v* ethylene glycol	0.03*M* of each halide	0.1*M* bicine/Trizma base pH8.5
B11	10% *w*/*v* PEG 4000, 20% *v*/*v* glycerol	0.03*M* of each halide	0.1*M* bicine/Trizma base pH8.5
B12	12.5% *w*/*v* PEG 1000, 12.5% *w*/*v* PEG 3350, 12.5% *v*/*v* MPD	0.03*M* of each halide	0.1*M* bicine/Trizma base pH8.5
C1	10% *w*/*v* PEG 20000, 20% *v*/*v* PEG MME 550	0.03*M* of each NPS	0.1*M* MES/imidazole pH6.5
C2	10% *w*/*v* PEG 8000, 20% *v*/*v* ethylene glycol	0.03*M* of each NPS	0.1*M* MES/imidazole pH6.5
C3	10% *w*/*v* PEG 4000, 20% *v*/*v* glycerol	0.03*M* of each NPS	0.1*M* MES/imidazole pH6.5
C4	12.5% *w*/*v* PEG 1000, 12.5% *w*/*v* PEG 3350, 12.5% *v*/*v* MPD	0.03*M* of each NPS	0.1*M* MES/imidazole pH6.5
C5	10% *w*/*v* PEG 20000, 20% *v*/*v* PEG MME 550	0.03*M* of each NPS	0.1*M* MOPS/HEPES-Na pH7.5
C6	10% *w*/*v* PEG 8000, 20% *v*/*v* ethylene glycol	0.03*M* of each NPS	0.1*M* MOPS/HEPES-Na pH7.5
C7	10% *w*/*v* PEG 4000, 20% *v*/*v* glycerol	0.03*M* of each NPS	0.1*M* MOPS/HEPES-Na pH7.5
C8	12.5% *w*/*v* PEG 1000, 12.5% *w*/*v* PEG 3350, 12.5% *v*/*v* MPD	0.03*M* of each NPS	0.1*M* MOPS/HEPES-Na pH7.5
C9	10% *w*/*v* PEG 20000, 20% *v*/*v* PEG MME 550	0.03*M* of each NPS	0.1*M* bicine/Trizma base pH8.5
C10	10% *w*/*v* PEG 8000, 20% *v*/*v* ethylene glycol	0.03*M* of each NPS	0.1*M* bicine/Trizma base pH8.5
C11	10% *w*/*v* PEG 4000, 20% *v*/*v* glycerol	0.03*M* of each NPS	0.1*M* bicine/Trizma base pH8.5
C12	12.5% *w*/*v* PEG 1000, 12.5% *w*/*v* PEG 3350, 12.5% *v*/*v* MPD	0.03*M* of each NPS	0.1*M* bicine/Trizma base pH8.5
D1	10% *w*/*v* PEG 20000, 20% *v*/*v* PEG MME 550	0.02*M* of each alcohol	0.1*M* MES/imidazole pH6.5
D2	10% *w*/*v* PEG 8000, 20% *v*/*v* ethylene glycol	0.02*M* of each alcohol	0.1*M* MES/imidazole pH6.5
D3	10% *w*/*v* PEG 4000, 20% *v*/*v* glycerol	0.02*M* of each alcohol	0.1*M* MES/imidazole pH6.5
D4	12.5% *w*/*v* PEG 1000, 12.5% *w*/*v* PEG 3350, 12.5% *v*/*v* MPD	0.02*M* of each alcohol	0.1*M* MES/imidazole pH6.5
D5	10% *w*/*v* PEG 20000, 20% *v*/*v* PEG MME 550	0.02*M* of each alcohol	0.1*M* MOPS/HEPES-Na pH7.5
D6	10% *w*/*v* PEG 8000, 20% *v*/*v* ethylene glycol	0.02*M* of each alcohol	0.1*M* MOPS/HEPES-Na pH7.5
D7	10% *w*/*v* PEG 4000, 20% *v*/*v* glycerol	0.02*M* of each alcohol	0.1*M* MOPS/HEPES-Na pH7.5
D8	12.5% *w*/*v* PEG 1000, 12.5% *w*/*v* PEG 3350, 12.5% *v*/*v* MPD	0.02*M* of each alcohol	0.1*M* MOPS/HEPES-Na pH7.5
D9	10% *w*/*v* PEG 20000, 20% *v*/*v* PEG MME 550	0.02*M* of each alcohol	0.1*M* bicine/Trizma base pH8.5
D10	10% *w*/*v* PEG 8000, 20% *v*/*v* ethylene glycol	0.02*M* of each alcohol	0.1*M* bicine/Trizma base pH8.5
D11	10% *w*/*v* PEG 4000, 20% *v*/*v* glycerol	0.02*M* of each alcohol	0.1*M* bicine/Trizma base pH8.5
D12	12.5% *w*/*v* PEG 1000, 12.5% *w*/*v* PEG 3350, 12.5% *v*/*v* MPD	0.02*M* of each alcohol	0.1*M* bicine/Trizma base pH8.5
E1	10% *w*/*v* PEG 20000, 20% *v*/*v* PEG MME 550	0.03*M* of each ethylene glycol	0.1*M* MES/imidazole pH6.5
E2	10% *w*/*v* PEG 8000, 20% *v*/*v* ethylene glycol	0.03*M* of each ethylene glycol	0.1*M* MES/imidazole pH6.5
E3	10% *w*/*v* PEG 4000, 20% *v*/*v* glycerol	0.03*M* of each ethylene glycol	0.1*M* MES/imidazole pH6.5
E4	12.5% *w*/*v* PEG 1000, 12.5% *w*/*v* PEG 3350, 12.5% *v*/*v* MPD	0.03*M* of each ethylene glycol	0.1*M* MES/imidazole pH6.5
E5	10% *w*/*v* PEG 20000, 20% *v*/*v* PEG MME 550	0.03*M* of each ethylene glycol	0.1*M* MOPS/HEPES-Na pH7.5
E6	10% *w*/*v* PEG 8000, 20% *v*/*v* ethylene glycol	0.03*M* of each ethylene glycol	0.1*M* MOPS/HEPES-Na pH7.5
E7	10% *w*/*v* PEG 4000, 20% *v*/*v* glycerol	0.03*M* of each ethylene glycol	0.1*M* MOPS/HEPES-Na pH7.5
E8	12.5% *w*/*v* PEG 1000, 12.5% *w*/*v* PEG 3350, 12.5% *v*/*v* MPD	0.03*M* of each ethylene glycol	0.1*M* MOPS/HEPES-Na pH7.5
E9	10% *w*/*v* PEG 20000, 20% *v*/*v* PEG MME 550	0.03*M* of each ethylene glycol	0.1*M* bicine/Trizma base pH8.5
E10	10% *w*/*v* PEG 8000, 20% *v*/*v* ethylene glycol	0.03*M* of each ethylene glycol	0.1*M* bicine/Trizma base pH8.5
E11	10% *w*/*v* PEG 4000, 20% *v*/*v* glycerol	0.03*M* of each ethylene glycol	0.1*M* bicine/Trizma base pH8.5
E12	12.5% *w*/*v* PEG 1000, 12.5% *w*/*v* PEG 3350, 12.5% *v*/*v* MPD	0.03*M* of each ethylene glycol	0.1*M* bicine/Trizma base pH8.5
F1	10% *w*/*v* PEG 20000, 20% *v*/*v* PEG MME 550	0.02*M* of each monosaccharide	0.1*M* MES/imidazole pH6.5
F2	10% *w*/*v* PEG 8000, 20% *v*/*v* ethylene glycol	0.02*M* of each monosaccharide	0.1*M* MES/imidazole pH6.5
F3	10% *w*/*v* PEG 4000, 20% *v*/*v* glycerol	0.02*M* of each monosaccharide	0.1*M* MES/imidazole pH6.5
F4	12.5% *w*/*v* PEG 1000, 12.5% *w*/*v* PEG 3350, 12.5% *v*/*v* MPD	0.02*M* of each monosaccharide	0.1*M* MES/imidazole pH6.5
F5	10% *w*/*v* PEG 20000, 20% *v*/*v* PEG MME 550	0.02*M* of each monosaccharide	0.1*M* MOPS/HEPES-Na pH7.5
F6	10% *w*/*v* PEG 8000, 20% *v*/*v* ethylene glycol	0.02*M* of each monosaccharide	0.1*M* MOPS/HEPES-Na pH7.5
F7	10% *w*/*v* PEG 4000, 20% *v*/*v* glycerol	0.02*M* of each monosaccharide	0.1*M* MOPS/HEPES-Na pH7.5
F8	12.5% *w*/*v* PEG 1000, 12.5% *w*/*v* PEG 3350, 12.5% *v*/*v* MPD	0.02*M* of each monosaccharide	0.1*M* MOPS/HEPES-Na pH7.5
F9	10% *w*/*v* PEG 20000, 20% *v*/*v* PEG MME 550	0.02*M* of each monosaccharide	0.1*M* bicine/Trizma base pH8.5
F10	10% *w*/*v* PEG 8000, 20% *v*/*v* ethylene glycol	0.02*M* of each monosaccharide	0.1*M* bicine/Trizma base pH8.5
F11	10% *w*/*v* PEG 4000, 20% *v*/*v* glycerol	0.02*M* of each monosaccharide	0.1*M* bicine/Trizma base pH8.5
F12	12.5% *w*/*v* PEG 1000, 12.5% *w*/*v* PEG 3350, 12.5% *v*/*v* MPD	0.02*M* of each monosaccharide	0.1*M* bicine/Trizma base pH8.5
G1	10% *w*/*v* PEG 20000, 20% *v*/*v* PEG MME 550	0.02*M* of each carboxylic acid	0.1*M* MES/imidazole pH6.5
G2	10% *w*/*v* PEG 8000, 20% *v*/*v* ethylene glycol	0.02*M* of each carboxylic acid	0.1*M* MES/imidazole pH6.5
G3	10% *w*/*v* PEG 4000, 20% *v*/*v* glycerol	0.02*M* of each carboxylic acid	0.1*M* MES/imidazole pH6.5
G4	12.5% *w*/*v* PEG 1000, 12.5% *w*/*v* PEG 3350, 12.5% *v*/*v* MPD	0.02*M* of each carboxylic acid	0.1*M* MES/imidazole pH6.5
G5	10% *w*/*v* PEG 20000, 20% *v*/*v* PEG MME 550	0.02*M* of each carboxylic acid	0.1*M* MOPS/HEPES-Na pH7.5
G6	10% *w*/*v* PEG 8000, 20% *v*/*v* ethylene glycol	0.02*M* of each carboxylic acid	0.1*M* MOPS/HEPES-Na pH7.5
G7	10% *w*/*v* PEG 4000, 20% *v*/*v* glycerol	0.02*M* of each carboxylic acid	0.1*M* MOPS/HEPES-Na pH7.5
G8	12.5% *w*/*v* PEG 1000, 12.5% *w*/*v* PEG 3350, 12.5% *v*/*v* MPD	0.02*M* of each carboxylic acid	0.1*M* MOPS/HEPES-Na pH7.5
G9	10% *w*/*v* PEG 20000, 20% *v*/*v* PEG MME 550	0.02*M* of each carboxylic acid	0.1*M* bicine/Trizma base pH8.5
G10	10% *w*/*v* PEG 8000, 20% *v*/*v* ethylene glycol	0.02*M* of each carboxylic acid	0.1*M* bicine/Trizma base pH8.5
G11	10% *w*/*v* PEG 4000, 20% *v*/*v* glycerol	0.02*M* of each carboxylic acid	0.1*M* bicine/Trizma base pH8.5
G12	12.5% *w*/*v* PEG 1000, 12.5% *w*/*v* PEG 3350, 12.5% *v*/*v* MPD	0.02*M* of each carboxylic acid	0.1*M* bicine/Trizma base pH8.5
H1	10% *w*/*v* PEG 20000, 20% *v*/*v* PEG MME 550	0.02*M* of each amino acid	0.1*M* MES/imidazole pH6.5
H2	10% *w*/*v* PEG 8000, 20% *v*/*v* ethylene glycol	0.02*M* of each amino acid	0.1*M* MES/imidazole pH6.5
H3	10% *w*/*v* PEG 4000, 20% *v*/*v* glycerol	0.02*M* of each amino acid	0.1*M* MES/imidazole pH6.5
H4	12.5% *w*/*v* PEG 1000, 12.5% *w*/*v* PEG 3350, 12.5% *v*/*v* MPD	0.02*M* of each amino acid	0.1*M* MES/imidazole pH6.5
H5	10% *w*/*v* PEG 20000, 20% *v*/*v* PEG MME 550	0.02*M* of each amino acid	0.1*M* MOPS/HEPES-Na pH7.5
H6	10% *w*/*v* PEG 8000, 20% *v*/*v* ethylene glycol	0.02*M* of each amino acid	0.1*M* MOPS/HEPES-Na pH7.5
H7	10% *w*/*v* PEG 4000, 20% *v*/*v* glycerol	0.02*M* of each amino acid	0.1*M* MOPS/HEPES-Na pH7.5
H8	12.5% *w*/*v* PEG 1000, 12.5% *w*/*v* PEG 3350, 12.5% *v*/*v* MPD	0.02*M* of each amino acid	0.1*M* MOPS/HEPES-Na pH7.5
H9	10% *w*/*v* PEG 20000, 20% *v*/*v* PEG MME 550	0.02*M* of each amino acid	0.1*M* bicine/Trizma base pH8.5
H10	10% *w*/*v* PEG 8000, 20% *v*/*v* ethylene glycol	0.02*M* of each amino acid	0.1*M* bicine/Trizma base pH8.5
H11	10% *w*/*v* PEG 4000, 20% *v*/*v* glycerol	0.02*M* of each amino acid	0.1*M* bicine/Trizma base pH8.5
H12	12.5% *w*/*v* PEG 1000, 12.5% *w*/*v* PEG 3350, 12.5% *v*/*v* MPD	0.02*M* of each amino acid	0.1*M* bicine/Trizma base pH8.5

**Table 2:** The 47 PDB-derived ligands selected to formulate MORPHEUS MPD is (*RS*)-2-methyl-2,4-pentanediol.

Ligand	Residue ID	No. of structures
(*RS*)-Tartaric acid	TAR, TLA	113
1,2-(*RS*)-Propanediol	PGR, PGO	41
1,3-Propanediol	PDO	7
1,4-Butanediol	BU1	11
1,6-Hexanediol	HEZ	19
1-Butanol	1BO	7
2-Propanol	IPA, IOH	174
Acetate anion	ACT, ACY, ACE	1890
Ammonium cation	NH4, NH3, NH2	582
Bicine	BCN	11
Bromide anion	BR	120
Calcium cation	CA	3959
Chloride anion	CL	2842
Citrate anion	FLC, CIT	384
D-Galactose	GLA, GAL	86
D-Glucose	GLC, BGC	206
Diethylene glycol	PEG	209
DL-Alanine	ALA, DAL	35
DL-Lysine	LYS, DLY	36
DL-Serine	SER, DSN	38
D-Mannose	MAN, BMA	178
D-Xylose	XYP, XYL	33
Ethylene glycol	EDO	1081
Fluoride anion	F	16
Formic acid	FMT	267
Glycerol	GOL	2884
Glycine	GLY	50
HEPES	EPE	201
Imidazole	IMD	154
Iodide anion	IOD	178
L-Fucose	FUC, FUL	62
L-Glutamic acid	GLU	28
Magnesium cation	MG	3991
MES	MES	315
MOPS	MPO	21
MPD	MRD, MPD	504
*N*-Acetyl-D-glucosamine	NAG	1150
Nitrate anion	NO3	156
Oxamic acid	OXM	17
Pentaethylene glycol	1PE	91
Phosphate anion	PO4, PI, 2HP	1687
Potassium cation	K	720
Sodium cation	NA	1926
Sulfate anion	SO4	5793
Tetraethylene glycol	PG4	194
Triethylene glycol	PGE	107
Tris	TRS	334
		
Total No. of entries		32908

**Table 3:** Recipes for preparing the eight MORPHEUS additive mixes

Stock	Composition
Divalent cations	0.3*M* magnesium chloride, 0.3*M* calcium chloride
Halides	0.3*M* sodium fluoride, 0.3*M* sodium bromide, 0.3*M* sodium iodide
NPS	0.3*M* sodium nitrate, 0.3*M* disodium hydrogen phosphate, 0.3*M* ammonium sulfate
Alcohols	0.2*M* 1,6-hexanediol, 0.2*M* 1-butanol, 0.2*M* (*RS*)-1,2-propanediol, 0.2*M* 2-propanol, 0.2*M* 1,4-butanediol, 0.2*M* 1,3-propanediol
Ethylene glycols	0.3*M* diethyleneglycol, 0.3*M* triethyleneglycol, 0.3*M* tetraethyleneglycol, 0.3*M* pentaethyleneglycol
Monosaccharides	0.2 *M* D-glucose, 0.2*M* D-mannose, 0.2*M* D-galactose, 0.2*M* L-fucose, 0.2*M* D-xylose, 0.2*M* *N*-acetyl-D-glucosamine
Carboxylic acids	0.2 *M* sodium formate, 0.2*M* ammonium acetate, 0.2*M* trisodium citrate, 0.2*M* sodium potassium L-tartrate, 0.2*M* sodium oxamate
Amino acids	0.2 *M* sodium L-glutamate, 0.2*M* DL-alanine, 0.2*M* glycine, 0.2*M* DL-lysine HCl, 0.2*M* DL-serine

**Table 4:** Recipes for preparing the four MORPHEUS precipitant mixes

Composition	Frequency	Reference
20% *w*/*v* PEG 20000, 40% *v*/*v* PEG MME 550	35	Cordell *et al.* (2003 ▶); Leonard *et al.* (2004 ▶); Selmer *et al.* (2006 ▶)
20% *w*/*v* PEG 8000, 40% *v*/*v* ethylene glycol	3	Teo *et al.* (2006 ▶)
20% *w*/*v* PEG 4000, 40% *v*/*v* glycerol	12	Low Lwe (2006 ▶)
25% *w*/*v* PEG 3350, 25% *w*/*v* PEG 1000, 25% *v*/*v* MPD	0	Not published

**Table:** 

pH	1*M* MES (ml)	1*M* imidazole (ml)
6.1	36.0	14.0
6.3	33.5	16.5
6.5	30.6	19.4
6.7	27.5	22.5
6.9	25.0	25.0

**Table:** 

pH	1*M* MOPS (ml)	1*M* HEPES-Na (ml)
7.1	34.5	15.5
7.3	30.0	20.0
7.5	25.9	24.1
7.7	22.1	37.9
7.9	17.7	32.3

**Table:** 

pH	1*M* bicine (ml)	1*M* Trizma base (ml)
8.1	35.6	14.4
8.3	31.7	18.3
8.5	26.7	23.3
8.7	21.2	28.8
8.9	15.0	35.0

**Table 6:** Details and results of the crystallization trials for 16 samples using MORPHEUS TEN 200 is a buffer containing 20m*M* Tris, 1m*M* ethylenediaminetetraacetic acid (EDTA), 1m*M* sodium azide and 200m*M* sodium chloride. In the Source column, LMB refers to the MRC Laboratory of Molecular Biology, Cambridge, England, Hutchison to the Hutchison/MRC Research Centre, Cambridge, England, and CPE to the Centre for Protein Engineering, Cambridge, England.

Symbol	Protein	Concentration (mgml^1^)	Molecular weight (kDa)	Source	Preparation/reference	Hits (well numbers)	Photo (Fig. 2 ▶)
TriUB-D	Triubiquitin complex	7.0	29.6	LMB, Yogesh Kulathu	Manuscript submitted	F01, F04, H01, H04	*a*, *b*
PI3K-I	Pi3-kinase 110delta with inhibitors	4.5	107.0	LMB, Alex Berndt	Manuscript submitted	C03, C04	*c*
Scc3	Cohesin subunit	10.0	47.0	LMB, Jan Lwe	To be published	H07	*d*
PBD	Plk1 polo-box domain	8.7	27.2	Hutchison, Ana J. Narvaez	Garcia-Alvarez *et al.* (2007 ▶)	B05, D05, D09, E05, F05, F09	*e*
PBD-P	Plk1 polo-box domain with compound	8.7	27.2	Hutchison, Ana J. Narvaez	To be published	D04	*f*
DivIVA	Tropomyosin	19.2	12.7	LMB, Marian Oliva	Manuscript in preparation	D07, F07	*g*
D1-D2	Sm protein complex	16.2	26.9	LMB, Chris Oubridge	Kambach *et al.* (1999 ▶)	G01	*h*
ParR	Chromosome partitioning	16.0	14.6	LMB, Jeanne Salje	Mller-Jensen *et al.* (2007 ▶)	G10, G11	*i*
CRY	P53 domain	6.5	27.0	CPE, Joel Kaar Nicolas Basse	Joerger *et al.* (2006 ▶)	D09, E09, G01, G05, G08, G09, G12, H09	*j*
BAR	BAR domain	6.0	29.0	LMB, Helen Kent	Peter *et al.* (2004 ▶)	A02, C04, C08, C12, G04, G08, G12	*k*
PAK4G	FtsK gamma domain	11.0	7.8	LMB, Jan Lwe	Sivanathan *et al.* (2006 ▶)	A01, A05	*l*
ScVps25	ESCRT II subunit	10.8	23.6	LMB, Olga Perisic	Wernimont Weissenhorn (2004 ▶)	A03, A06, B10, C05, C09, E03, E06, E07, E10, F03, F06, F07, F10	*m*, *n*
Ran	Ran GTPase	10.0	24.5	LMB, Danguole Ciziene	Stewart *et al.* (1998 ▶)	G04	*o*
CNVA	Concanavalin A	7.0	26.5	Sigma, L7647	Dissolved in TEN 200 pH 8.5	D02, D06, E02, E06, E10, H02, H06	*p*
THM	Thaumatin	30.0	22.0	Sigma, T7638	Dissolved in deionized water	G01, G05, G09	*q*
LYS	Lysozyme	10.0	14.4	Sigma, L6876	Dissolved in deionized water	A05, A08, B06, B07, C05, C06, C08, D05, E05, G05, G07, H05	*r*
